# The dual effect of social ties on COVID-19 spread in Japan

**DOI:** 10.1038/s41598-021-81001-4

**Published:** 2021-01-15

**Authors:** Timothy Fraser, Daniel P. Aldrich

**Affiliations:** 1 Political Science Department, Northeastern University, 960A Renaissance Park, 360 Huntington Avenue, Boston, MA 02115-5000 USA; 2 Public Policy and Urban Affairs and Director of Security and Resilience Program, Department of Political Science, Northeastern University, 215H Renaissance Park, 360 Huntington Avenue, Boston, MA 02115 USA

**Keywords:** Infectious diseases, Respiratory tract diseases, Risk factors, Natural hazards

## Abstract

We investigate why some communities experience worse COVID-19 outcomes than others. Past studies have linked the resilience of communities against crisis to social vulnerability and the capacity of local governments to provide public goods and services like health care. Disaster studies, which frequently examine the effect of social ties and mobility, may better help illuminate the current spread of COVID-19. We analyze Japan’s 47 prefectures from February 12 to August 31 using 62,722 individual confirmed cases of COVID-19, paired with daily tallies of aggregate Facebook user movement among neighborhoods. Controlling for mobility levels, health care systems, government finance, gender balance, age, income, and education levels of communities, our analysis indicates that areas with strong linking social ties see no or far lower levels of COVID-19 case rates initially. However, case fatality rates rise in such communities once the disease enters as they lack horizontal (bonding) ties which can mitigate its health impacts. We anticipate this study to be a starting point for broader studies of how social ties and mobility influence COVID-19 outcomes worldwide along with shining a light on how different types of social relationships play different roles as a crisis or disaster progresses.

## Introduction

As the pandemic claims more than 1,000,000 lives globally, many observers seek to understand differing levels of COVID-19 outcomes at a variety of levels of analysis. Since early 2020, scholars have looked at the capacity of health care systems, the density of residents, and the social vulnerability of communities to crisis as potential explanations for variation in pandemic consequences in communities, states, and nations. But social ties—a key factor in studies of disaster mitigation and recovery^1,2^—have largely remained absent from the conversation.

This study of Japan’s 47 prefectures (the equivalent to US states) over a 6 months period in 2020 finds that, controlling for demographic, mobility, health care system, and political factors, communities with strong linking social capital initially see measurably lower levels of COVID-19 cases (or none at all). After the pandemic reaches a certain threshold, however, those same vertical connections correlate with increased COVID-19 case rates and fatality rates; each finding was valid within 95% confidence intervals, as we discuss below. In the same way that past research has documented a dark side to social capital^3–5^, so too our study sees that strong connections can correlate with positive and negative social outcomes at different moments in a crisis.

This study makes several contributions to the literature on disasters, pandemics, and COVID19. First, while past studies have linked social capital^4,6^ and social vulnerability^7,8^ to disaster outcomes, this study applies a social infrastructure lens to the COVID-19 pandemic outcome. We find that communities with weak linking social capital are less likely to face COVID outbreaks (p < 0.05, SI Table 2), but areas with strong linking social capital tend to see worse outbreaks if they are already showing cases (p < 0.05, SI Table 3), as they typically have lower levels of horizontal, bonding ties (p < 0.001, SI Table 4).

Second, going beyond papers on the disease which have used only census data or other pre-pandemic indicators, this study leverages up-to-date, daily Facebook and Google Android user mobility data as key mediating variables to discern the relationships between social ties and COVID-19 case rates, building on a literature on the role of mobility in crises^9,10^. Geographic mobility has been linked to the rise of past diseases, such as avian flu^11^ and SARS^12^, as well as COVID-19^13,14^. Here, our results indicate that trust in government health directives may be affecting people’s mobility and risk-threshold differently depending on civil society—state relations during the pandemic (as highlighted later in Figs. 6 and 7).

Third, this study highlights that while health care system capacity is vital to reducing the spread of pandemics^15^, individual citizens and communities’ participation in public health efforts are vital to ensuring widespread adoption of new health behaviors. This builds on past findings from SARS^16,17^ and Ebola^18^, highlighting that bridging (p < 0.001, SI Table 4) and linking social ties (p < 0.05, SI Table 2) are especially key in slowing the initial impact.

Fourth, where many studies of the pandemic have used a snapshot approach to capture a single moment in the disease, we follow the advice of social scientists to see how time interacts with the variables over a longer period^19^. Rather than assuming that our variables of interest interact with outcomes in the same way at different points over time, we leverage a longer term perspective—nearly half a year—to better understand those changing relationships. We see that while deeper reservoirs of vertical social connections initially are protective for Japanese prefectures (p < 0.05, SI Table 2), these ties—and their negative correlation with horizontal bonding connections (p < 0.001, SI Table 4)—end up highly correlated with fatality rates among the infected (p < 0.05, SI Table 3). Viewing social ties at a single spot in time would lead to spurious inference.

Finally, where many studies of social capital’s role in public policy have emphasized its positive outcomes, here we see evidence that it may, like the Roman god Janus, have two faces^3,20^. Early in the pandemic, strong vertical ties help communities reduce the likelihood of the disease entering the community (p < 0.05, SI Table 2). But later, once the disease enters, those same high levels of linking ties correlate with higher levels of case mortality (p < 0.05, SI Table 3). Hence, we see social capital’s dark side at work.

## Literature review

This study examines why some Japanese prefectures saw more (or fewer) new cases of COVID-19 and why others encountered higher (or lower) death rates during the pandemic. Recent scholarship highlights that COVID-19 spreads through contact with aerosolized droplets from persons carrying the virus, facilitated by coughing and sneezing^21^. On average, it takes 5 days to develop symptoms, with a range of 1–14 days^22^. Tracking infection rates has been problematic, as some states (such as the US and Japan) were slow to begin testing cases and have failed to contact trace^23^. Further, many people spread the virus asymptomatically^24^. Based on infections reported already, we can examine variation in infection rates and in death rates among communities.

**Figure 1:**
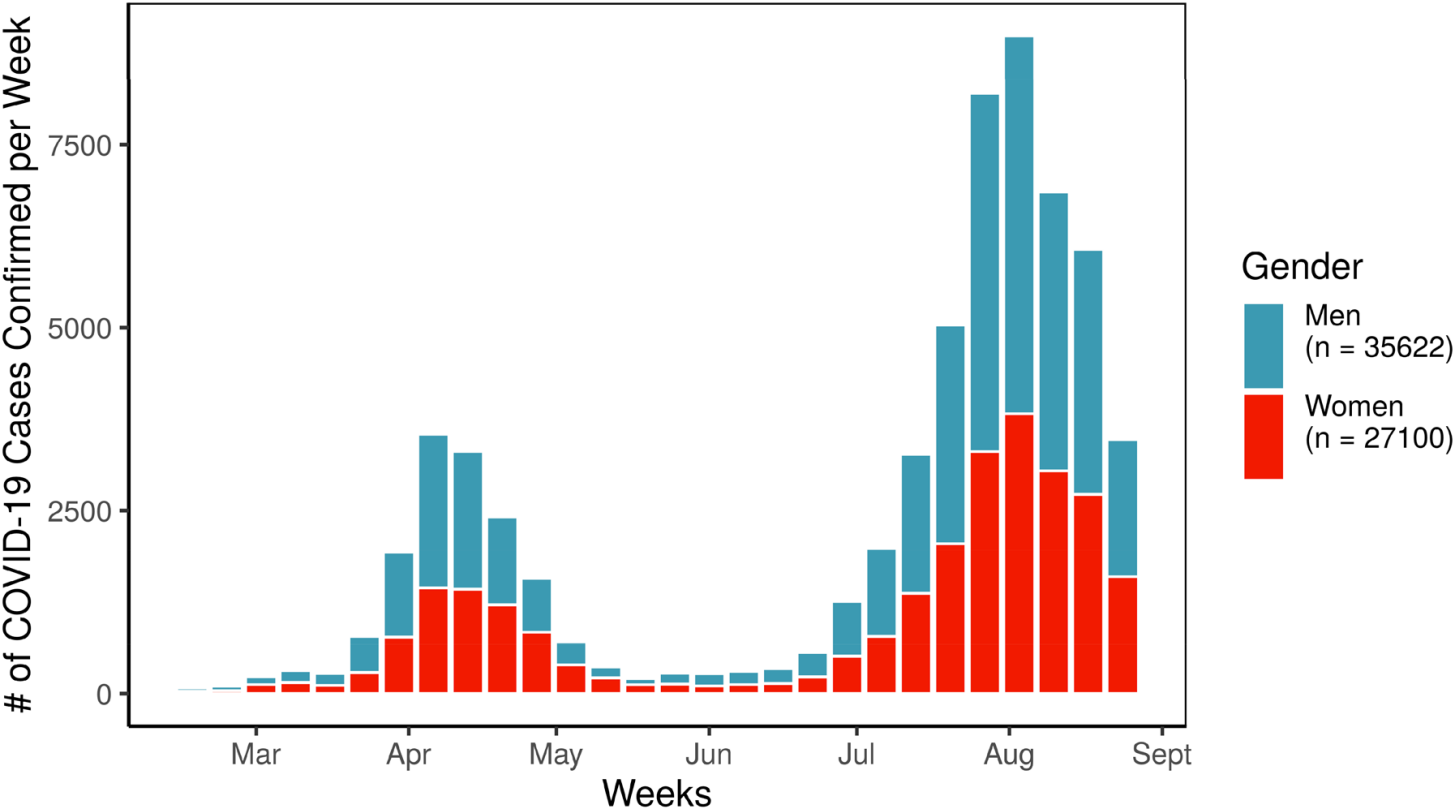
COVID-19 cases confirmed over time in JAG japan sample.

One reason for higher rates of infection might come from of citizens’ behaviors and mobility during the pandemic^13,14^. Communities have adopted physical distancing at varying rates; many Japanese prefectures and religious organizations did not close key institutions until early April^23,25^. For example, according to reports from the Ministry of Health, Labor, and Welfare, the number of confirmed cases of COVID-19 per week saw a dip from May through late June, when school shutdowns and changes in individual behavior limited the spread of the virus, but cases spiked again between July and August, especially among men (See Fig. 1).

**Figure 2:**
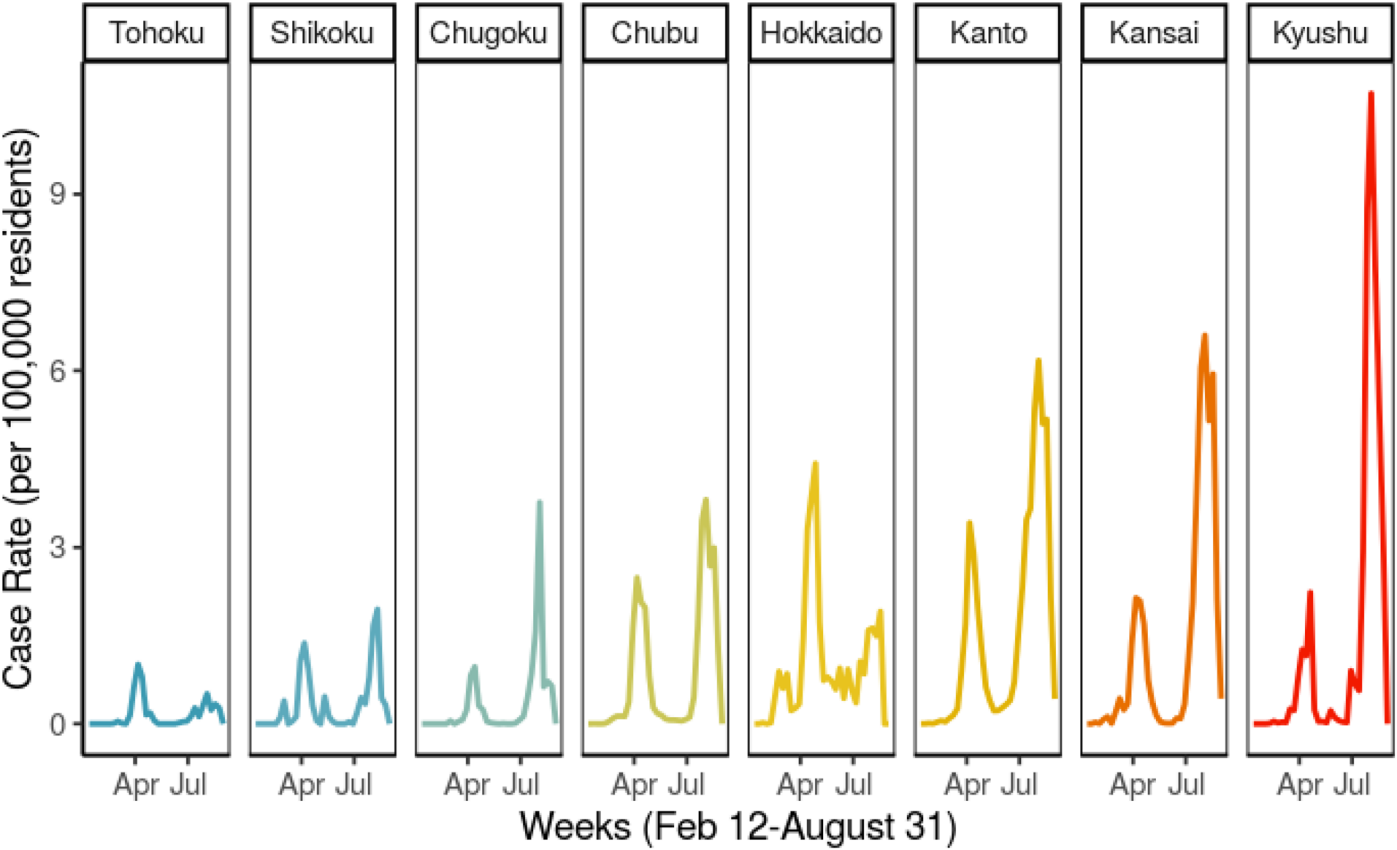
Changing average weekly case rates by region.

Similarly, the northern prefecture of Hokkaido saw a high share of cases early on, while Aichi Prefecture in the Chubu Region developed and contained clusters of infections gradually instead. The greater Tokyo metropolitan area, known as Kansai, and the southern island of Kyushu have become COVID hotspots more recently (See Fig. 2). Communities where residents still move between neighborhoods frequently often encounter higher case rates^11,12^. Similarly, communities which already have developed cases are more likely to see spread due to exponential rates of infection.

However, some communities see especially high rates of infection and death. In the US, African American neighborhoods with high shares of low-income residents in New York City and the city of Flint, Michigan, have seen disproportionately high infection rates^26^. These communities have greater shares of socially vulnerable populations, such as residents who are elderly, women, single parents, unemployed, in poverty, or racial, religious, or ethnic minorities. These populations tend to see worse outcomes both from initial disasters and long-term recovery processes^8,27^, because they are financially constrained from seeking help and have faced institutionalized discrimination in the past. Further, such populations may be in front line jobs such as delivery, security, manufacturing, and hospitality and unable to work from home, increasing the likelihood of interactions with infected persons.

Yet some vulnerable communities manage better outcomes from crisis than others due to the *capacity* of governments to provide better quality response^28–30^. In the case of COVID-19, some communities had better funded governments that purchased necessary materials and had more doctors, nurses, hospitals, and clinics available to serve new waves of patients^15^. Meanwhile, others struggled to provide similar levels of care for populations.

Finally, even vulnerable communities with weak government and health care capacity can respond better to crisis if they have strong social networks to rely on. Disaster scholars find that strong social capital—social ties that residents use for physical, financial, and social support in times of crisis—are powerful interventions that boost community resilience^4^. Scholars found this after the 1995 Kobe Earthquake^3,31^, the 1995 heat wave in Chicago^32^, the 2011 disaster in Japan^2,6,33^, and after Hurricanes Katrina^34^, Matthew, and Harvey^1,35,36^ in the US.

Social capital comes in three forms: bonding, bridging, and linking social ties. *Bonding ties* connect members of the same social groups, like family members, neighbors, and members of co-ethnic or co-religious groups, and help those groups survive crisis, but can lead to hoarding of resources. *Bridging ties* connect members of different social groups, like unions, nonprofits, and volunteer organizations, facilitating civic engagement^37^, reducing ethnic violence^38^, and providing mutual support across different social groups^3^. Finally, *linking ties* connect residents to local, regional, and national officials, helping them access key public goods they might not otherwise receive, and are frequently measured using levels of trust in government^2,39,40^.

In the case of COVID-19, broader, more diverse social networks boost the spread of quality information on how to keep community members from contracting the virus. Past studies of epidemics found that information from trusted personal ties was more effective in changing health behaviors than centralized information campaigns^16–18^. We hypothesize that areas characterized primarily by strong bonding social ties might see higher levels of COVID spread, as they would lack diverse sources of information from experts and outsiders. In contrast, we hypothesize that deeper reservoirs of bridging and linking ties might reduce COVID rates, facilitating the spread of quality information, as residents who trust their officials and different social groups would be more likely to implement physical distancing, personal protective equipment, and hand washing. However, when the disease is rampant in a community, stronger bonding ties can provide assistance for sick residents, helping them reach medical personnel along with food, water, and psychological support for what may be a long battle with the pandemic. As we discovered, once the disease has entered a prefecture, communities with stronger vertical ties but fewer local, horizontal ties found themselves with higher rates of mortality.

## Results

This study studied weekly infection and case fatality rates for Japan’s 47 prefectures based on anonymized records of 62,722 individual cases of COVID-19 from February 10 to August 31. These records were distributed to the public by Japan’s Ministry of Health, Labor, and Welfare and compiled by JAG Japan^41^. We analyzed this data at the aggregate, prefecture-week level and at the individual level. In each model, we tested the effect of social capital, including bonding, bridging, and linking social capital, drawing from new social capital indices (cf. Fraser^6^ for details on this framework), case rates in the previous week, and change in residents’ movement patterns in the previous week. We use a lag in our data because it takes 5 days, on average, for an infected person to show symptoms. We assessed mobility using aggregate level data from Facebook’s Data for Good project, as well as Google Android user mobility data to confirm that our results were consistent across different samples of resident mobility. Meanwhile, each model controlled for the capacity of health care systems, government finances, gender balance, age, income, and education levels of communities, alongside further demographic controls. Finally, since social processes might change as communities adapt to the new pandemic, we modeled these infection rates using weekly fixed effects, compared with weekly random effects and first-order autocorrelation. Our modeling techniques, including proxies used in these models, are discussed in depth in the “Methods” section at the conclusion of this article.

### Aggregate analysis

**Figure 3:**
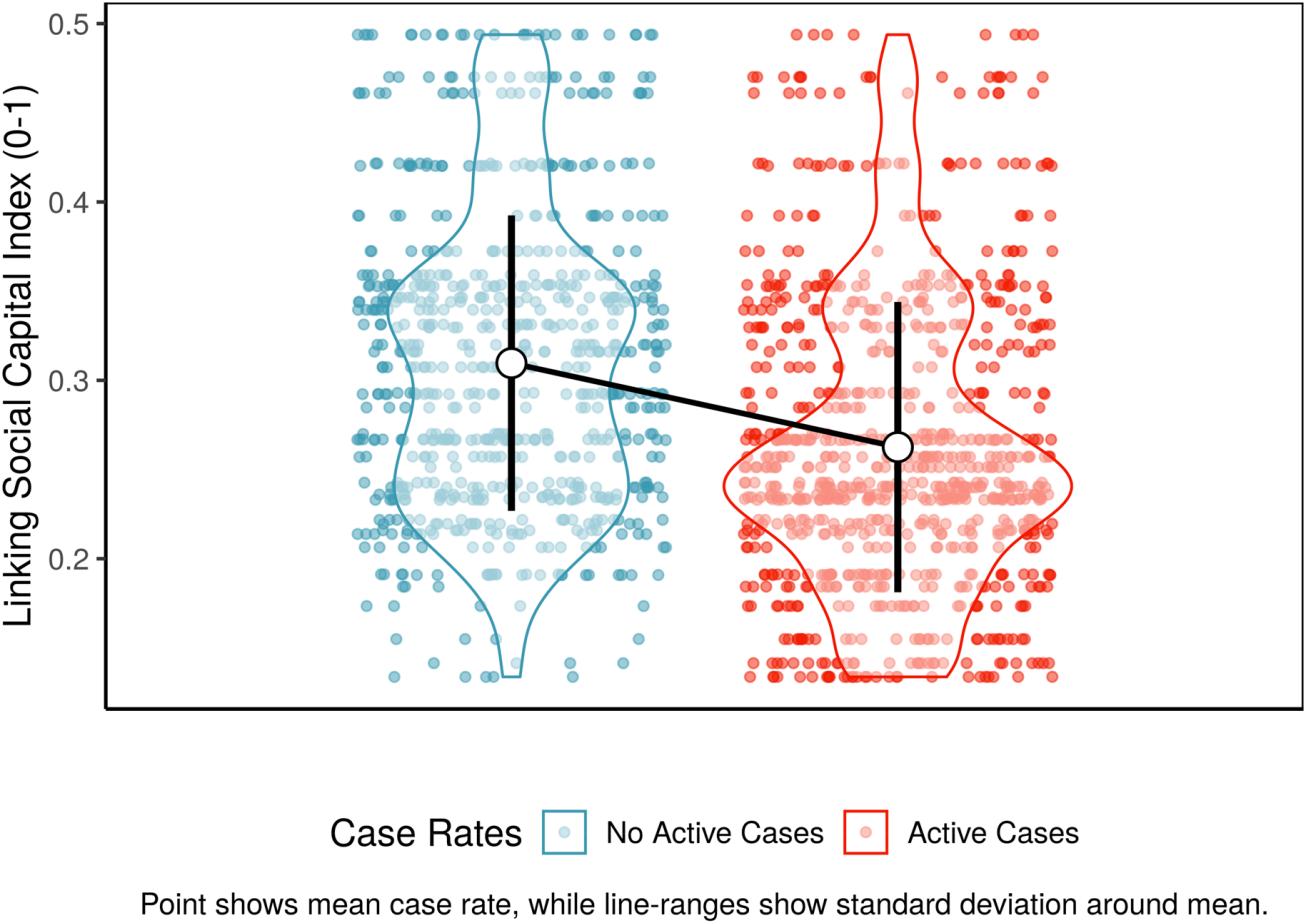
Linking social capital vs. COVID outbreaks.

This analysis finds strong evidence that linking social capital is closely associated with initially low COVID-19 case rates but eventually higher fatality rates. First, we modeled why some prefectures encountered more cases per week of COVID-19 per 100,000 residents than others using an ordinary least squares model. To confirm these results, we also analyzed why some prefectures encountered *any* cases per week of COVID-19 (0/1), using a logit model. In both cases, we found that communities with stronger linking social capital scores were more likely to see *lower* case rates of COVID-19, or none at all (SI Table 2, p < 0.05). Figure 3 highlights this trend descriptively, showing that prefecture-weeks with no active cases tended to have higher social capital than prefecture-weeks with *any* active cases.

However, when we focused on prefectures where COVID-19 had taken root, leading to at least one confirmed case, we saw a different relationship between COVID-19 spread and linking social capital. First, we modeled why some prefectures with at least one case encountered more cases per 100,000 residents per week than others. To complement this glimpse of COVID-19 hotspots, we also analyzed why some prefectures-weeks encountered higher case fatality rates than others. Outbreak areas demonstrated a strong *positive* relationship between linking social capital and both case fatality rates and case rates (p < 0.05).

**Figure 4:**
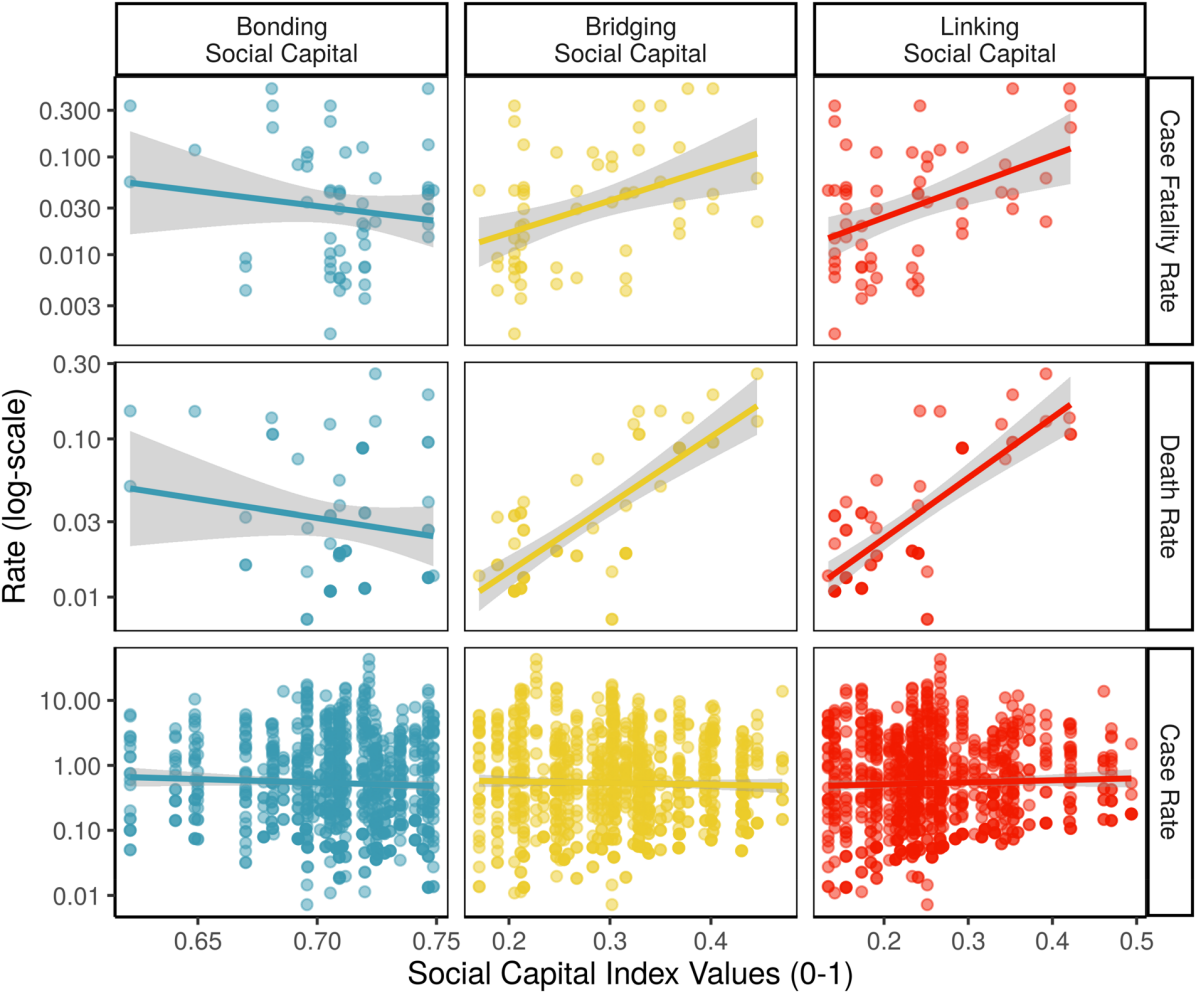
Social capital vs. COVID case rates and case fatality rates (in prefectures with active COVID cases).

Descriptively, when we zoom into places with a least one confirmed case, we can see this as well in Fig. 4, which highlights that prefecture-weeks with strong linking social capital see higher case fatality rates. After controlling for bonding and bridging social capital in our model, the only type of connections which remained statistically significant was linking social ties (p < 0.05, SI Table 3). We also see in the figure the negative relationship between bonding social capital and case fatality rates, again demonstrating that these two types of social capital do not overlap, but rather are negatively correlated. Communities with stronger vertical ties have weaker horizontal ones (with a Pearson’s r of − 0.32).

**Figure 5:**
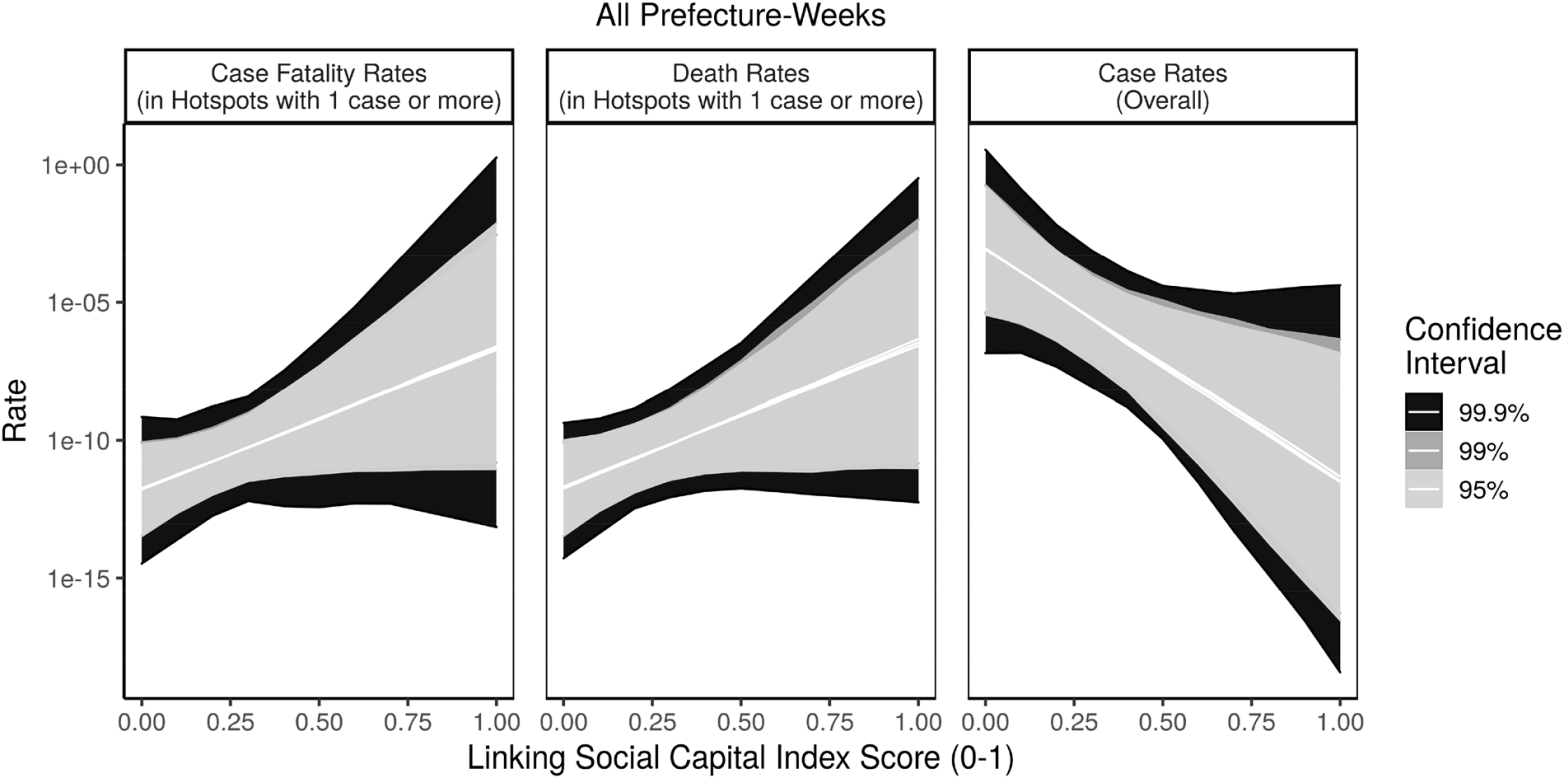
Divergent effects of linking social capital.

Figure 5 highlights linking social capital’s negative relationship with COVID-19 spread overall (right panel), paired with its positive association with COVID-19 case fatality rates (left panel) and death rates (center panel) in hotspots with at least 1 case. To generate Fig. 5, we simulated the effect of linking social capital on case fatality rates compared with overall case rates over 1000 simulations. We kept all of the other variables in our models at their means and allowed linking social capital levels to vary^42^. These simulations depict the expected rate of each outcome, highlighting projections for which we have 95, 99, and 99.9% confidence intervals, respectively.

We provide several potential explanations for this Janus-like set of divergent trends with regards to linking social capital. First, residents in prefectures with stronger linking social capital are more likely to trust recommendations from local government and health authorities, helping limit the spread of those clusters. Initially, then, they successfully reduced the infection rate. However, as others have reported^23,43^, the Japanese central government’s reactions to COVID fell far short of public health experts’ recommendations, with limited changes to transportation, workplaces, and more. In other words, strong linking social capital and trust in government could be wasted if government officials do not take appropriate action to curb the spread of COVID-19.

Next, hotspot communities with connections to decision makers had fewer connections horizontally to neighbors, kin, and friends (as shown by the effect of bonding social capital in SI Table 4, p < 0.001). Once COVID-19 entered the prefecture, individuals getting sick had fewer resources and networks on which to draw for assistance, whether physical (getting to a medical facility) or psychological (being supported over what may be a long period of time on a ventilator in an ICU). As scholars have argued, communities cannot simultaneously grow all three types of social connections due to time and resource constraints^3,20^. Those with deeper reservoirs of one type (in this case, linking ties) found themselves lacking another (bonding).

**Figure 6:**
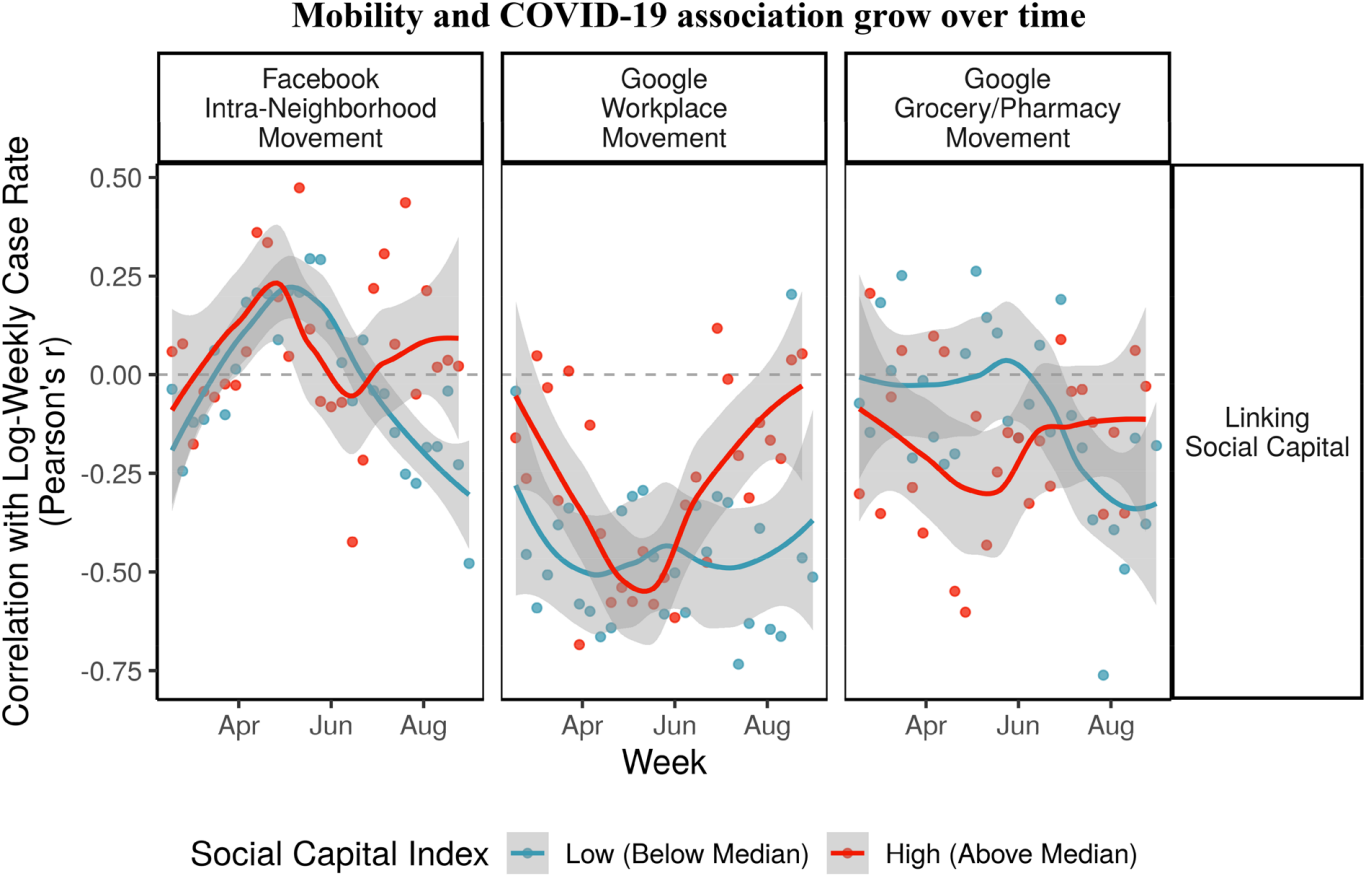
Prefectures with strong linking social capital see Mobility and COVID-19 association grow over time.

In fact, we can tangibly connect linking social capital to changing mobility patterns. Figure 6 shows the changing correlation between weekly case rates (log-transformed) and change in mobility patterns the week before, for prefectures with linking social capital *above* (*red*) and *below* (*blue*)* the median.* We plotted loess smoothed curves to show the overall change week to week.

The correlations differ slightly due to different measures of mobility but a general pattern can be seen. While communities with strong linking social capital saw *low or negative correlations between case rates and mobility* early on, that pattern changed after June, when they saw increasingly positive or neutral associations. A possible explanation for this is that prefectures with stronger linking social capital had trust in government messages that they could resume normal social and economic activities leading to people to lower their guards and go out more. As mobility patterns increased, case rates in the subsequent week increased as well.

### Individual analysis

To triangulate these findings, we also examined anonymized records of 62,722 individuals reported to have contracted COVID-19. We predicted the level of linking social capital in each individual’s prefecture based on their age, gender, and the same prefecture-level controls as applied in the aggregate analysis. We found that residents from prefectures that saw especially high mobility from Facebook users were likely to be from a prefecture with high linking social capital. This trend persisted among both COVID-19 survivors and those with fatal cases.

**Figure 7:**
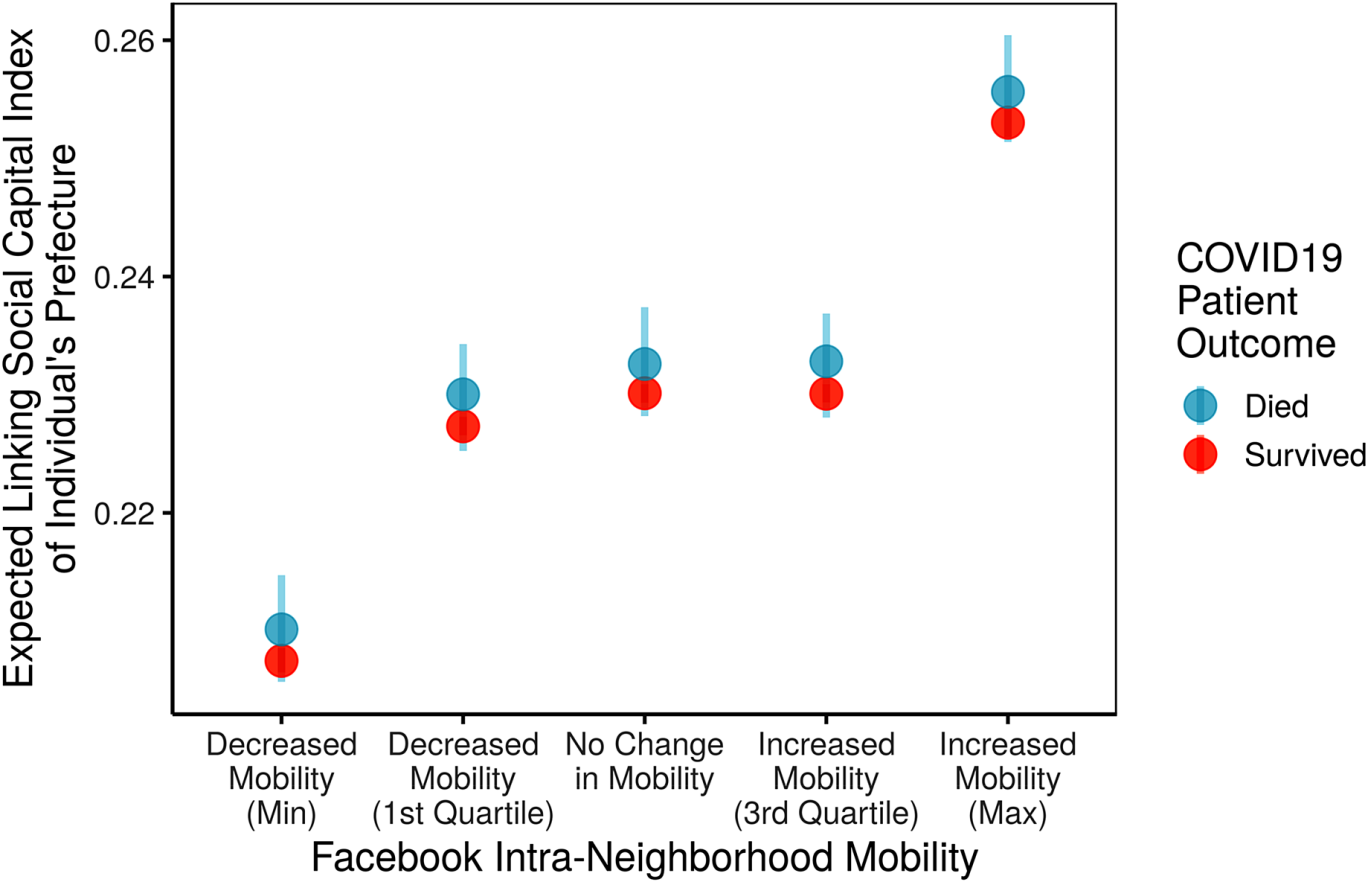
Higher linking social capital expected from individuals in high mobility places.

This trend is highlighted in Fig. 7, which used 1000 simulations of this individual level model to show the expected level of linking social capital in a prefecture given five different levels of mobility, within a 95% confidence interval. Letting mobility vary, we held all other variables in our models at their mean and mode when carrying out these simulations. Our model also projected that people who died were almost always from places with higher linking social capital, while survivors were almost always from prefectures with low linking social capital.

## Discussion

In summary, we see preliminary evidence that social ties and mobility patterns are shaping the spread of COVID-19 among Japanese prefectures. Our study finds vertical, linking social capital was related to an initial decline in COVID-19 spread, except in hotspots, where places with strong linking social capital suffered most. We found evidence this was because of the negative correlation between higher linking social capital and bonding social ties. Here we see the trade-offs evident with different categories of social connections^20^. This two-sided association of linking social capital is worrisome, for it raises questions about quality of governance in COVID-19 hotspots.

This study also found the surprising secondary result that bonding social capital was associated with a decrease in COVID spread and fatalities (Fig. 4). Similarly, COVID patients from prefectures with higher linking social capital tended to have weaker bonding social capital, but stronger bridging social capital (SI Table 4). We expected the opposite, because closely bonded communities might limit the spread of key information, while bridging communities might facilitate the spread of public health information. One interpretation of this is that associational participation, a component of bridging social capital, might have helped spread COVID, while insular neighborhood ties did not. A second interpretation is that because of wide media coverage about COVID19, bonding social capital’s insulating effect on information flow was less relevant, leaving just its beneficial effects, such as trust. If so, trust in government messages about COVID might have a larger effect on residents’ behaviors than insular social networks. Future studies should investigate this bonding social capital effect further.

Similarly, it is possible that prefectures with strong linking social capital adopted different policies than peer prefectures, such that linking social capital was an intermediary variable, rather than a direct determinant of COVID outcomes. Future studies should investigate further the potential intervening role of linking social capital in COVID policy outcomes.

One challenge of inferring the effect of social ties on infection is that the Japanese government has been widely criticized for testing too few residents over the last 6 months. Critics might argue that we only observed that linking ties were related to infection rates *because* prefectures with stronger social ties tend to have better quality governance, and those prefectures used those resource and trust networks to identify and test more people. However, this explanation is not sufficient. If communities with stronger linking ties test *more*, we would expect the effect of linking social ties over time to produce a false *positive.* However, we find the opposite. This lends credence to our hypothesis that, despite limited testing in Japan, linking social ties are important correlates of COVID-19 spread over time. A second challenge of examining social ties is that communities have changed as COVID-19 unfolded. As residents spent more time with family, commuted less, and companies reduced normally grueling hours, the monthly suicide rate in Japan dropped precipitously by 20% in April^44^. As a result, overall models of social behavior during this period eclipse key changing trends over time. However, this study compensated for changing social conditions by modeling the effect of each week separately using fixed or random effects.

Further, one advantage of this research is that it controls for the tendency of residents to move among different neighborhoods, using aggregate tallies of Facebook user movement. Data from Facebook users serve as a relatively accurate means of measuring movement, as similar shares of users ages 20–59 and male and female use Facebook^45^. See the “Methods” for further information on Facebook demographics. However, to confirm these effects, we repeated our aggregate analysis using aggregate tallies of Google Android phone user movement as well, producing similar results. It seems unlikely that two different samples of mobile phone movement would coincidentally reveal the same association with linking social capital. Further, two groups underrepresented in mobile phone samples (children and the elderly) also tend to be consistently less mobile, meaning that they would likely not affect our measures of change in mobility.

Finally, this study relied on an aggregate data of all COVID19 cases reported by the Ministry of Health, Labor, and Welfare (MHLW), tallied by JAG Japan. This study did not employ a random sample, but we would expect that because we drew from the full universe of known, reported COVID cases, that these results would closely follow the true pattern of COVID spread in Japan. Since this dataset cover the full known universe of COVID cases during the study period, p-values are not strictly necessary, but we relied on them here to indicate trends that are especially strong such that we can be more confident that they did not occur by chance.

Our individual level analysis draws from over 62,000 anonymized COVID patients in Japan, and this allowed us to directly adjust for patient age, gender, and whether or not they survived. However, to protect patients’ privacy, the MHLW did not release other key demographic data; this is a limitation of our aggregate analysis. Future studies should investigate to what degree individual level traits such as education, income level, social connectivity, and trust in government affected COVID spread; qualitative studies in particular would be helpful to identify under what circumstances bonding, bridging, or linking social capital might aid these individuals.

In summary, this study finds that social ties are an important correlate with COVID-19 spread, drawing on the case of Japanese prefectures from February 12 to August 31. Though communities with strong linking social ties may have seen less spread overall, those communities now in COVID-19 hotspots tend to have stronger reservoirs of linking social capital. Future studies should investigate at the individual level how trust in government affects residents’ adoption of new physical distancing behaviors, mask wearing, and reduced mobility. Future studies should examine whether residents who affiliate with the party in power are adopting these behaviors or not. By channeling trust in government to quality behavioral interventions, we hope this research can improve our capacity to respond not just to disasters but also pandemics.

## Methods

This preliminary study examines why some Japanese prefectures saw higher case rates and case fatality rates from COVID-19 than others. Using publicly available reports of COVID cases from the Ministry of Health, Labor, and Welfare, this study analyses how social ties shape the spread of COVID-19, while adjusting for the effects of human mobility, prior infections, social vulnerability to crisis, health care capacity, governance capacity, and demographics. This data stretches from February 12 to August 31, was collected by the Japanese firm JAG Japan, and made available to the public^41^. We took these individual reports and generated both aggregate and individual level models to discern the relationship between social capital and COVID-19 outbreaks.

*First*, we modeled why some prefectures encountered more cases per week of COVID-19 per 100,000 residents than others, using an ordinary least squares model (OLS). Model results are displayed in Supplementary Information (SI) Table 2 (left side). To confirm these results, we also modeled why some prefectures encountered *any* cases per week of COVID-19 (0/1), using a logit model (See SI Table 2, right side). *Second*, we modeled why some prefecture-weeks encountered higher case fatality rates than others, using OLS models. Week-to-week models are more reliable than daily models, because local governments may report cases a day or two late. Results are shown in SI Table 3. *Third*, to validate our findings (about linking social capital), we used these individual reports and OLS models to examine why some individual with confirmed cases of COVID hailed from prefectures with higher levels of linking social capital than others. These results are highlighted in SI Table 4. This three-pronged approach helps contextualize *when* key social processes affect COVID-19 spread the most.

### Key variables

This analysis employs several key predictors. To model social capital, we use new indices modeled after the indices by Kyne and Aldrich^46^, aggregated to the prefectural level. As an initial analysis, we model just social capital, while subsequent analyses replace the social capital index with subindices for bonding, bridging, and linking social capital. All indices range from 0 to 1, where 1 denotes the most social capital, and 0 signifies the least. While these indices are discussed further in the referenced literature^6,46^, we describe them below briefly.

These indices drew on 21 total indicators, listed in entirety in SI Table 1. The bonding social capital index represents the mean of seven measures of how similar members of a community are, in terms of nationality, religious, education, employment, employment levels by gender, age, and communication capacity. These include (1) fractionalization by nationality and (2) by religion, (3) the negative absolute different between those college educated and those educated up through elementary school, (4) fractionalization by gender employment rates, (5) the absolute different between the share of workers employed and unemployed, (6) the share of residents below age 65, and (7) the number of television service contracts per capita. The index draws heavily on fractionalization measures, introduced by Alesina et al.’s work on racial and ethnic fractionalization in communities^47^.

The bridging social capital index represents the mean of eight measures of how interconnected members of different social groups are through associational ties. These include civil social participation and norm adoption, measured through (1) volunteer participation rates and (2) voter turnout rates in national elections and (3) prefectural elections. These also include neighborhood ties, measured with the rate of (4) community centers and (5) libraries per capita; union ties, measured by (6) the number of unions per capita; civic organizations, measured by (7) the number of nonprofit organizations per capita; and religious ties, measured with 8) the number of religious organizations per capita.

Finally, the linking social capital index is measured using six measures. Local government linkage is measured using (1) the number of local government employees per capita; prefectural government linkage is measured using (2) the number of prefectural police per capita and (3) prefectural government employees per capita. National government linkage is measured using the (4) share of the vote for the ruling party in national elections, while political linkages were supplemented with (5) the number of prefectural assembly representatives per capita and (6) the share of the vote for the ruling party in prefectural elections.

These indicators for social capital were validated as correlated appropriately with known social capital relationships with disaster outcomes, including public works spending, outmigration rates, and death rates^6^.

Next, to represent mobility, we ran models twice, once using Facebook mobility measures, once using Google Android mobility measures. We calculated the change in total Facebook users who moved between neighborhoods within prefectures since the start date of the analysis, lagged by 1 week. This is to account for the fact that it takes on average 5 days for COVID-19 spread to result in symptoms and new cases. Further, focusing on intra-prefectural, inter-neighborhood movement is a good way to capture increases or decreases in everyday mobility, which could put people in contact with COVID through their daily lives. This data was provided by Facebook’s Data for Good project.

We compared these measures with Google Android user data, provided by Google Mobility Reports. Google estimates for known places in Google Maps a baseline level of mobility pre-COVID, gathered from the median level of movement to places from the period January 3 to Feb 6, 2020. Then, they calculate the percent change in movement each day compared to that baseline, broken up into movement to specific kinds of places. These places include retail and recreation, groceries and pharmacies, parks, transit stations, workplaces, and residential. This analysis relied on workplace and grocery/pharmacy movement data, two distinct kinds of movement (whereas workplace, transportation, and residential movement are very colinear). For each week, we took the mean of daily percent changes in mobility to workplaces and groceries/pharmacies.

This study never had any contact with individual level Facebook or Google user data, but instead uses aggregated data provided by Facebook and Google. Any Facebook user data was collected by Facebook Data for Good according to Facebook's Data Use Policy, then aggregated to the neighborhood level to maintain individuals’ privacy, so that researchers never had contact with individual level data. This aggregate level data is regularly provided to humanitarian NGOs and research teams with data sharing agreements, and does not involve any sensitive data nor user data. This analysis is an observational study of aggregate-level Facebook and Google data, so no Institutional Review Board protocol was necessary.

Facebook users are a decent approximation of movement in the population; similar shares of users across age groups and gender use Facebook. According to a survey by Japan’s Ministry of Internal Affairs and Communications (MIAC) in 2019, 32.8% of Japanese reported using Facebook, compared with 17% of teens, 47% of users ages 20–29, 49% of users ages 30–39, 37% of users ages 40–49, 29% of users ages 50–59, and 14% of users ages 60–69. Rates of use among men and women were identical (33%)^45^. This gives us a highly detailed glimpse of movement within or between prefectures, helping us assess the effect of this movement on spread rates.

### Controls

This analysis also applied several control variables. First, we controlled for spread in urban areas using population density per 1000 persons. Next, to represent overall health conditions, we use the life expectancy of men in a prefecture (men have higher death rates from COVID). We also tallied the number of people with high risk health conditions, specifically diabetes, heart disease, or hypertension, per capita. (These are the health conditions available at the prefectural level).

Further, we control for health care capacity, because communities with better health care capacity might identify, quarantine, and treat affected patients faster. We use the total expenditures on health by municipalities or prefectural government per capita, in thousands of yen. Next, we also control for government capacity using the health of municipality budgets, represented by the ratio of revenues to expenditures.

Finally, we control for several demographic traits linked to social vulnerability. We control for age using the percentage of the population over age 65, for gender using the share of women in the population, for wealth using income per capita, for education using the share of university educated residents, and the stress of economic conditions using the unemployment rate. Several of these were collinear; to keep collinearity below a VIF score of 10, we split up age, income per capita, and education into six quantiles. These categorical measures still represent socioeconomic variation among these 47 prefectures with a high level of detail, while also removing problematic multicollinearity with might otherwise distort our estimates.

Since COVID-19 cases are only identified anonymously by prefecture, this is the highest level of detail available, but future studies may improve on this if municipal level case rates become available. Finally, when using the individual level data, we used the same array of predictors, but substituted prefectural gender and age measures for the actual reported gender and age of each individual.

### Models

This study generated 26 models of case rates and case fatality rates, listed in SI Tables 2–4. Each OLS model used a log transformation of case rates or case fatality rates as the dependent variable, to account for the considerable right skew of the data. Because our prefecture-week models (24 out of 26 models) occurred over time, we took additional steps to ensure that any statistically significant results were not just artifacts of heteroskedasticity, which is normal in time-series data. To verify this, we modeled each outcome three ways, first holding weeks as fixed effects, then second using random effects, then third using random effects with first order autocorrelation on the dependent variable, in case places with high case rates in 1 week led to higher case rates in the next week. Our fixed and random effects models also each contained as a predictor the outcome for the week prior, to adjust for potential autocorrelation through a time lag. We removed this predictor for our random effects models with first order autocorrelation, which serves the same purpose. We found consistent statistically significant associations with linking social capital across each model at the p < 0.05 or, in only a handful of cases, the p < 0.10 level. These results are highlighted in SI Tables 2 and 3. The fact that these reappeared across multiple modeling strategies *and* appeared in multiple descriptive visualizations gives us greater confidence that these are not spurious associations. As a robustness check, we repeated these models for death rates in SI Table 3, and found results quite consistent with case fatality rates. We draw from these models in Fig. 5.

Further, our models fit the data quite well. Each model of overall case rates explained at least 49% of the variation in weekly case rates of COVID-19. And while our models explained at least 65% of the variation in case rates in COVID hotspots, as well as at least 18% of variation in case fatality rates. (It does not surprise us that our models explain case rates better than case fatality rates, as there are numerous exogenous factors affecting mortality, which an individual level study could better capture).

Based on chi-squared intercept tests, all models fit better than an intercept model, with a statistically significant fit (p < 0.001). Multicollinearity problems were abated by keeping the average variance inflation factor below 10, a problematic level of multicollinearity, meaning that it does not affect the validity of the model. Each model depicts standardized coefficients, which describe the increase in case rates given an increase of one tenth of the range of that predictor. As a result, the size of effects can be compared across different variables to show which variable has the largest estimated effect on the outcome. For all models except SI Table 4, these effects are written as log-odds, because the dependent variable is either log-transformed in an OLS model or an actual logit model. Descriptive statistics for the aggregate and individual datasets are available in SI Tables 5 and 6.

### Ethics statements

All methods were carried out in accordance with relevant guidelines and regulations. This study never had any contact with individual level Facebook or Google user data, but instead uses aggregated data provided by Facebook and Google. Any Facebook user data were collected by Facebook Data for Good according to Facebook’s Data Use Policy, then aggregated to the neighborhood level to maintain individuals’ privacy, so that researchers never had contact with individual level data. This aggregate level data are regularly provided to humanitarian NGOs and research teams with data sharing agreements and do not involve any sensitive data nor user data. This analysis is an observational study of aggregate-level data, so no Institutional Review Board protocol was necessary.

## Supplementary Information

Supplementary Tables.

## Data Availability

The datasets generated and analysed during the current study, as well as all replication code, are available on the Harvard Dataverse repository (https://doi.org/10.7910/DVN/LA2VWS).
