# Supplementary material for: The dual effect of social ties on COVID-19 spread in Japan
Source: Sci Rep. 2021 Jan 15;11:1596. doi: 10.1038/s41598-021-81001-4 (PMC7811012; doi:10.1038/s41598-021-81001-4)

**Supplementary Information (SI) for Manuscript:**

**The Dual Effect of Social Ties on COVID-19 spread in Japan**

Timothy Fraser^1*^ & Daniel P. Aldrich^2^

**1.** Timothy Fraser, PhD Candidate, Political Science Department, Northeastern University

Address: 960A Renaissance Park, 360 Huntington Avenue, Boston, MA 02115-5000 USA

Email: [timothy.fraser.1@gmail.com](mailto:fraser.ti@husky.neu.edu)

* Corresponding author

**2.** Daniel P. Aldrich (Ph.D.), Professor of Political Science, Public Policy and Urban Affairs and Director of Security and Resilience Program, Northeastern University

Email: [daniel.aldrich@gmail.com](mailto:daniel.aldrich@gmail.com)

**Table of Contents**

- **SI Table 1:** Social Capital Index Indicators
- **SI Table 2:** Models of Case Rates per Prefecture-Week
- **SI Table 3:** Models of COVID-19 per prefecture-week in Hotspots with at least 1 case
- **SI Table 4:** Models of Prefectural Linking Social Capital per individual case of COVID-19
- **SI Table 5:** Descriptive Statistics (Aggregate Prefecture-Weeks Dataset)
- **SI Table 6:** Descriptive Statistics (Individual Dataset with Prefecture Traits)

**SI Table 1: Social Capital Index Indicators**

| **Index** | **Concept** | **Effect** | **Indicator** |
| --- | --- | --- | --- |
| Bonding  Social Capital | Nationality similarity | - | Nationality Fractionalization  (Japanese vs. Foreign Population) |
|  | Religious similarity | - | Fractionalization by Religious Minority (Religious Minorities vs. Non-Minority Residents) |
|  | Educational equality | - | Negative absolute difference between percentage of total population with college education and percentage of elementary school graduates |
|  | Gender employment similarity | - | Fractionalization of employment equality by gender (Women’s vs. Men's Employment Equality) |
|  | Employment equality | + | Absolute difference between % of employed and % of unemployed labor force |
|  | Communication capacity | + | television broadcast reception contracts per capita |
|  | Non-Elder population | + | % of total population below 65 years of age |
| Bridging  Social Capital | Social embeddedness:  civil society participation and norm adoption | + | Volunteer Participation Rate (over age 10)  Voter Turnout in Prefectural Elections  Voter Turnout in Lower House Elections |
|  | Social embeddedness:  neighborhood ties | + | Community Centers per capita  Libraries per capita |
|  | Social embeddedness:  union ties | + | Unions per capita |
|  | Civic Organizations | + | Nonprofit Organizations per capita |
|  | Religious organizations | + | Religious organizations per capita |
| Linking  Social Capital | Local Government Linkage | + | Local government employees per capita |
|  | Prefectural government linkage | + | Prefectural government employees per capita  Prefectural Police per capita |
|  | National  government linkage | + | % of vote for Ruling Party in House of Reps elections |
|  | Political Linkage:  political activities | + | Prefectural Assembly members per capita  % of vote for Ruling Party in Prefectural Elections |
| * Notes: Table borrowed from Fraser (2020) and lightly edited for clarity. | | | |

**SI Table 2: Models of Case Rates per Prefecture-Week**
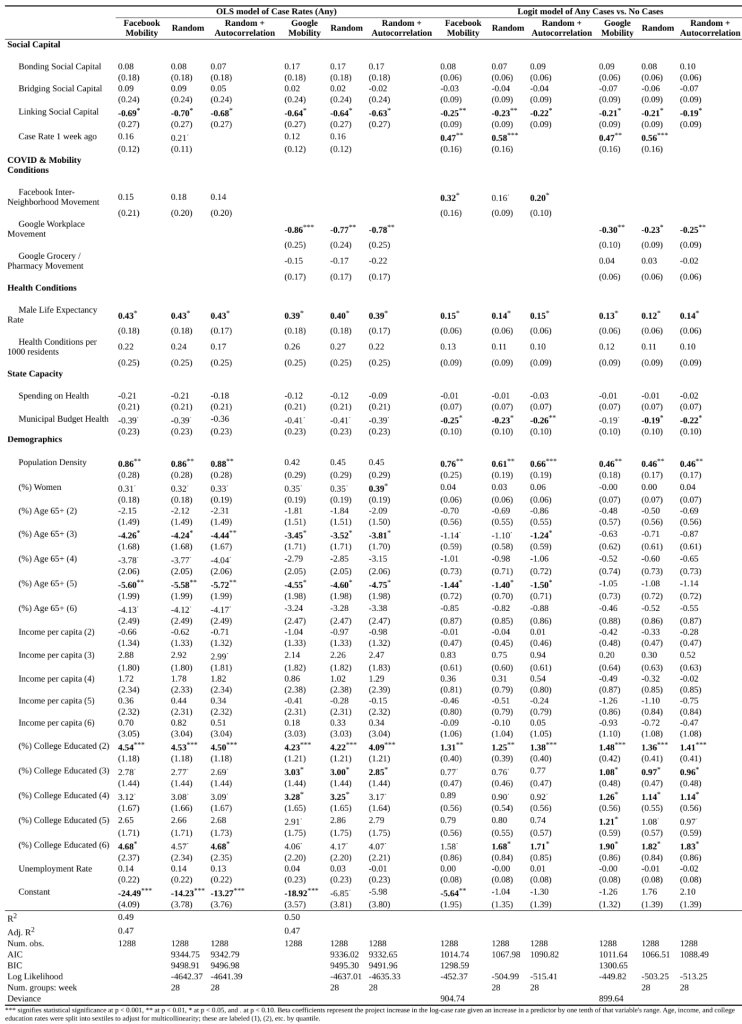


**SI Table 3: Models of COVID-19 per prefecture-week**

**in Hotspots with at least 1 case**


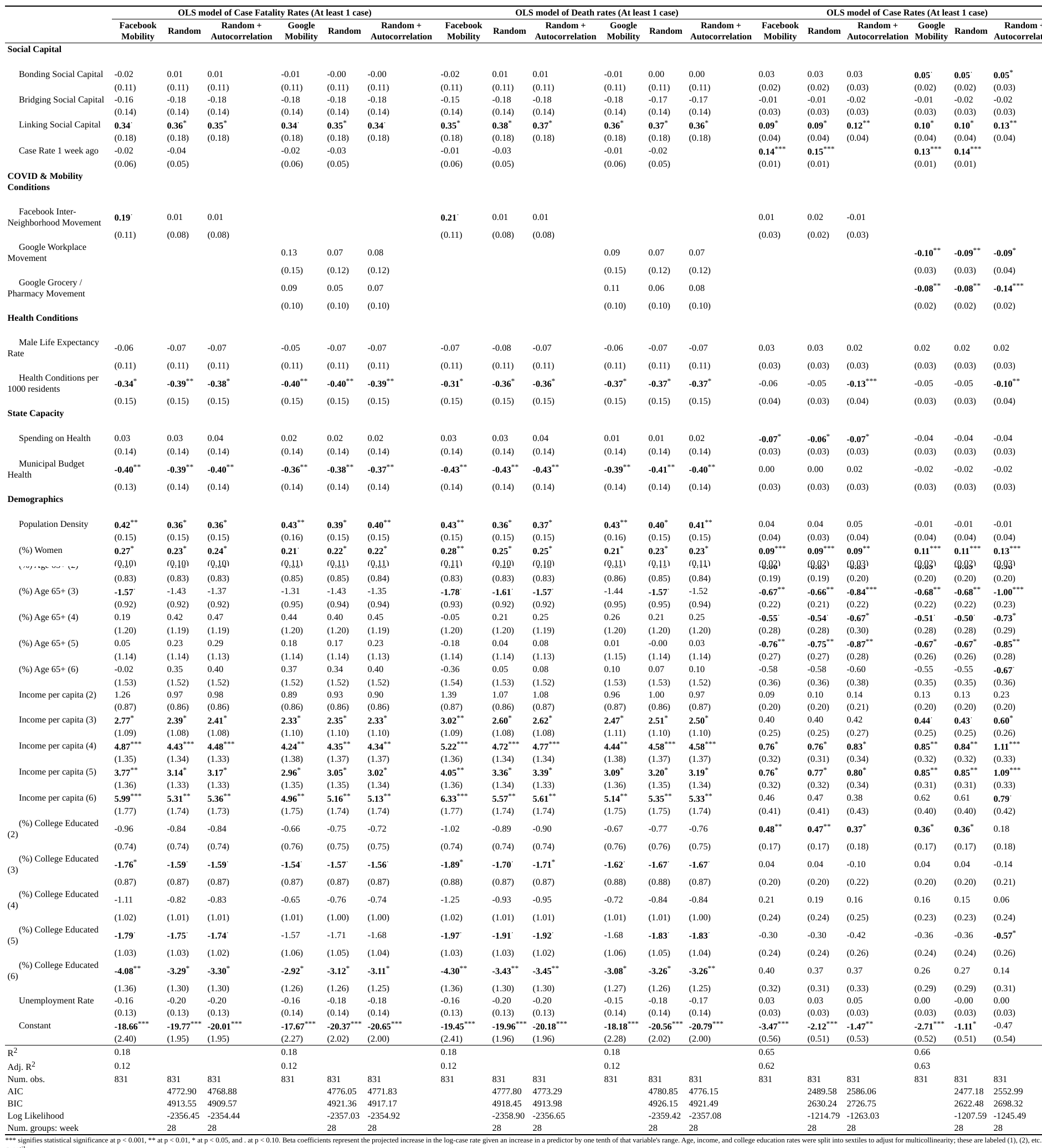


**SI Table 4: Models of Prefectural Linking Social Capital**

**per individual case of COVID-19**


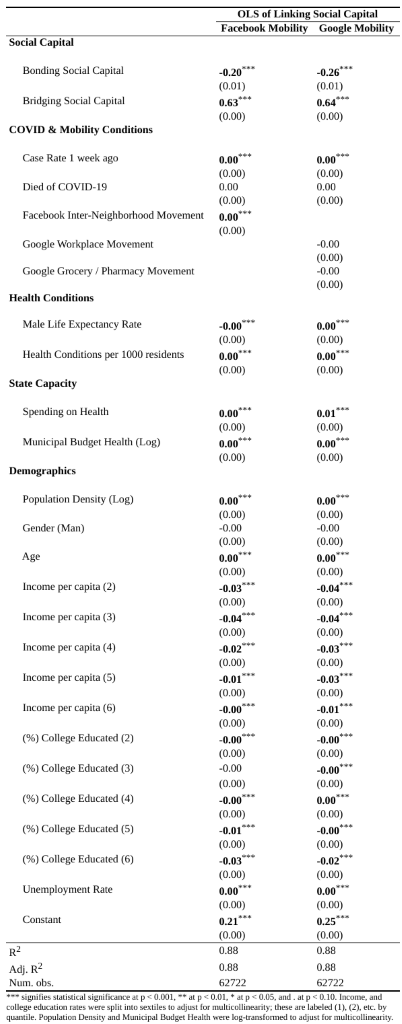


**SI Table 5: Descriptive Statistics (Aggregate Prefecture-Weeks Dataset)**


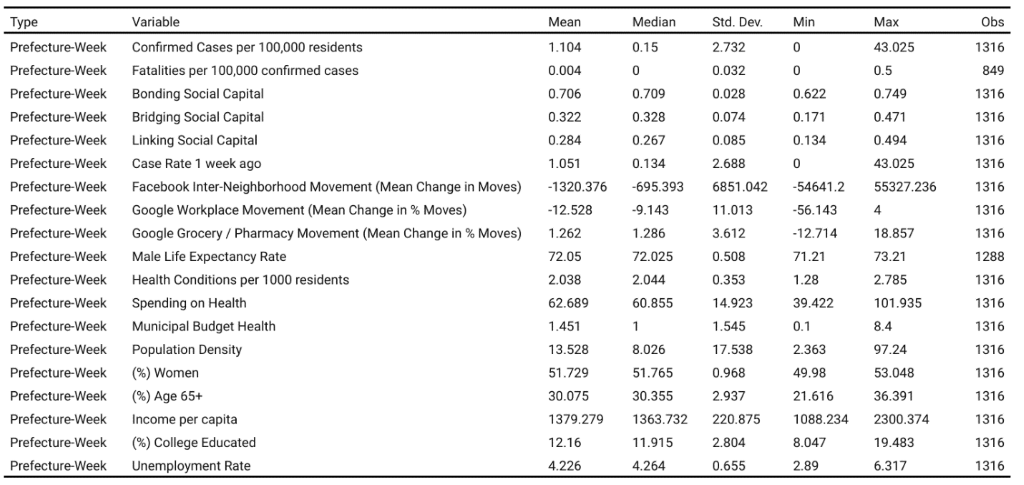


**SI Table 6: Descriptive Statistics (Individual Dataset with Prefecture Traits)**


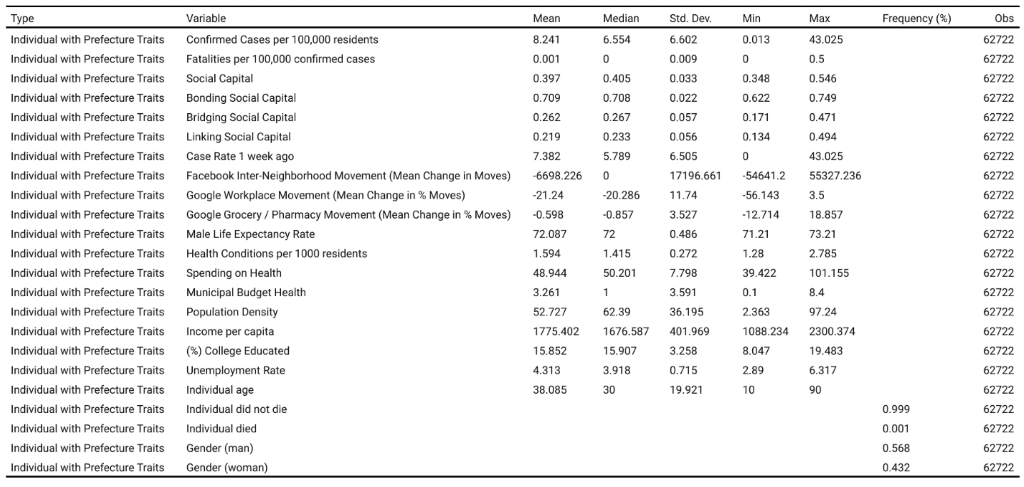

Supplement: Supplementary file 1 — Supplementary Tables. [file 41598_2021_81001_MOESM1_ESM.docx]
